# Bioactive Constituents of Ginger and Potential Mechanisms Underlying Its Anti-Rheumatoid Arthritis Effects: Integrated Efficacy Evaluation, Chemical Profiling, Network Pharmacology, and Molecular Dynamics Simulation

**DOI:** 10.3390/metabo16090661

**Published:** 2026-09-09

**Authors:** Hancheng Li, Jinwei Gan, Yuting Huang, Yangkai Wu, Chaohua Luo, Wenhua Liu, Hongwu Wang, Zhixian Mo

**Affiliations:** 1 Department of Pharmaceutical Engineering, School of Food and Pharmaceutical Engineering, Zhaoqing University, Zhaoqing 526061, China; lihancheng@zqu.edu.cn (H.L.); 18269651893@163.com (J.G.); 19102064352@163.com (Y.H.); ykwu661@163.com (Y.W.); 2 Department of Pharmacology of Chinese Medicine, School of Traditional Chinese Medicine, Southern Medical University, Guangzhou 510515, China; lchua2008@smu.edu.cn; 3 Key Laboratory for Research and Utilization of Southern Medicine, Zhaoqing University, Zhaoqing 526061, China; liuwenhua89745@163.com; 4 Risk Assessment Laboratory for Agricultural Product Quality and Safety, Ministry of Agriculture and Rural Development, Zhaoqing University, Zhaoqing 526061, China

**Keywords:** *Zingiber officinale*, rheumatoid arthritis, UPLC-Q-TOF-MS/MS, network pharmacology, adjuvant-induced arthritis, molecular docking

## Abstract

**Background/Objectives:** Rheumatoid arthritis (RA) is a chronic autoimmune inflammatory disease, and ginger contains phenolic constituents with anti-inflammatory potential. This study evaluated the antiarthritic efficacy of ginger ethanolic extract (GE) and explored its potential mechanisms through integrated pharmacological and computational approaches. **Methods:** GE was prepared by 70% ethanol reflux extraction and administered to adjuvant-induced arthritis (AIA) rats, with methotrexate as a positive control. Body weight; paw swelling; arthritis index; spleen index; and serum interleukin-6 (IL-6), interleukin-1β (IL-1β), tumor necrosis factor-α (TNF-α), and prostaglandin E2 (PGE2) were measured. Chemical profiling was performed by ultra-performance liquid chromatography–quadrupole time-of-flight tandem mass spectrometry (UPLC-Q-TOF-MS/MS), followed by network pharmacology, molecular docking, and molecular dynamics (MD) simulation. **Results:** GE attenuated paw edema, arthritis index elevation, splenic enlargement, and elevated serum inflammatory mediators in AIA rats. UPLC-Q-TOF-MS/MS annotated 38 constituents, mainly gingerols, shogaols, gingerdiones, gingerdiols, and related phenolic derivatives. Network analysis identified 217 overlapping ginger- and RA-associated targets, with TNF, IL6, and matrix metalloproteinase 9 (MMP9) prioritized among inflammatory and matrix-remodeling nodes. Docking and MD simulations supported stable predicted interactions for 8-gingerol-TNF and 6-gingerol-MMP9. **Conclusions:** GE showed antiarthritic activity in AIA rats. The integrated chemical, in vivo, and computational data suggest that ginger phenolics may modulate inflammatory mediators and candidate RA-related pathways; however, these mechanisms remain exploratory and require histological, tissue-level, and molecular validation.

## 1. Introduction

Rheumatoid arthritis (RA) is a chronic systemic autoimmune disorder characterized by persistent synovial inflammation, pannus formation, cartilage destruction, and progressive bone damage [1,2]. The initiation and progression of RA involve complex interactions among genetic susceptibility, environmental factors, and immune dysregulation. Persistent inflammatory signaling is a key driver of disease progression, in which tumor necrosis factor-α (TNF-α), interleukin-1β (IL-1β), and interleukin-6 (IL-6) promote inflammatory cell recruitment, fibroblast-like synoviocyte activation, and tissue injury [3,4,5]. In addition, cyclooxygenase-2 (COX-2)/prostaglandin E_2_ (PGE_2_) signaling, Janus kinase-signal transducer and activator of transcription (JAK-STAT), mitogen-activated protein kinase (MAPK), phosphatidylinositol 3-kinase-protein kinase B (PI3K-Akt), nuclear factor-κB (NF-κB) pathways, and matrix metalloproteinase (MMP)-mediated extracellular matrix degradation contribute to chronic inflammation and structural joint damage [6,7,8]. Current therapeutic approaches for RA, including nonsteroidal anti-inflammatory drugs, glucocorticoids, conventional disease-modifying antirheumatic drugs, biological agents, and targeted synthetic agents, have substantially improved disease management. However, limitations such as adverse effects, incomplete therapeutic responses, relapse after treatment withdrawal, and economic burden remain important challenges [9,10,11]. Therefore, natural products with anti-inflammatory, immunomodulatory, and joint-protective activities have attracted increasing interest as potential complementary strategies for RA intervention.

In traditional Chinese medicine (TCM), RA is commonly considered within the framework of Bi syndrome, which emphasizes the imbalance of qi and blood, invasion of pathogenic factors, and impaired nourishment of tendons and joints [12,13,14]. The multicomponent and multitarget characteristics of TCM provide a theoretical basis for investigating complex inflammatory diseases. Several medicinal herbs and formulas, including *Tripterygium wilfordii*, *Paeonia lactiflora*, Cinnamomi Ramulus, Duhuo Jisheng Decoction, and Simiao Yongan Decoction, have demonstrated potential regulatory effects on inflammatory and immune responses [15,16]. Ginger (*Zingiber officinale* Roscoe), widely used as both food and medicine, has a long history of application for pain relief and inflammatory disorders in TCM, Ayurveda, and Persian medicine [17,18]. Modern pharmacological studies have demonstrated that ginger exhibits anti-inflammatory, antioxidant, analgesic, gastroprotective, and immunomodulatory activities. Its major chemical constituents include gingerols, shogaols, gingerdiones, gingerdiols, volatile oils, polysaccharides, and other phenolic derivatives [19,20,21]. Among these compounds, 6-gingerol, 8-gingerol, 10-gingerol, and 6-shogaol have been considered important contributors to ginger-mediated anti-inflammatory effects through regulation of NF-κB-related signaling, inhibition of pro-inflammatory cytokine production, and modulation of oxidative stress responses [22,23]. Previous studies have reported that ginger-derived compounds can reduce TNF-α, IL-1β, IL-6, PGE_2_, and nitric oxide production, suggesting potential relevance to RA-associated inflammation [24,25,26]. However, the chemical basis and potential molecular mechanisms underlying ginger-mediated antiarthritic effects remain incompletely defined.

**Figure 1:**
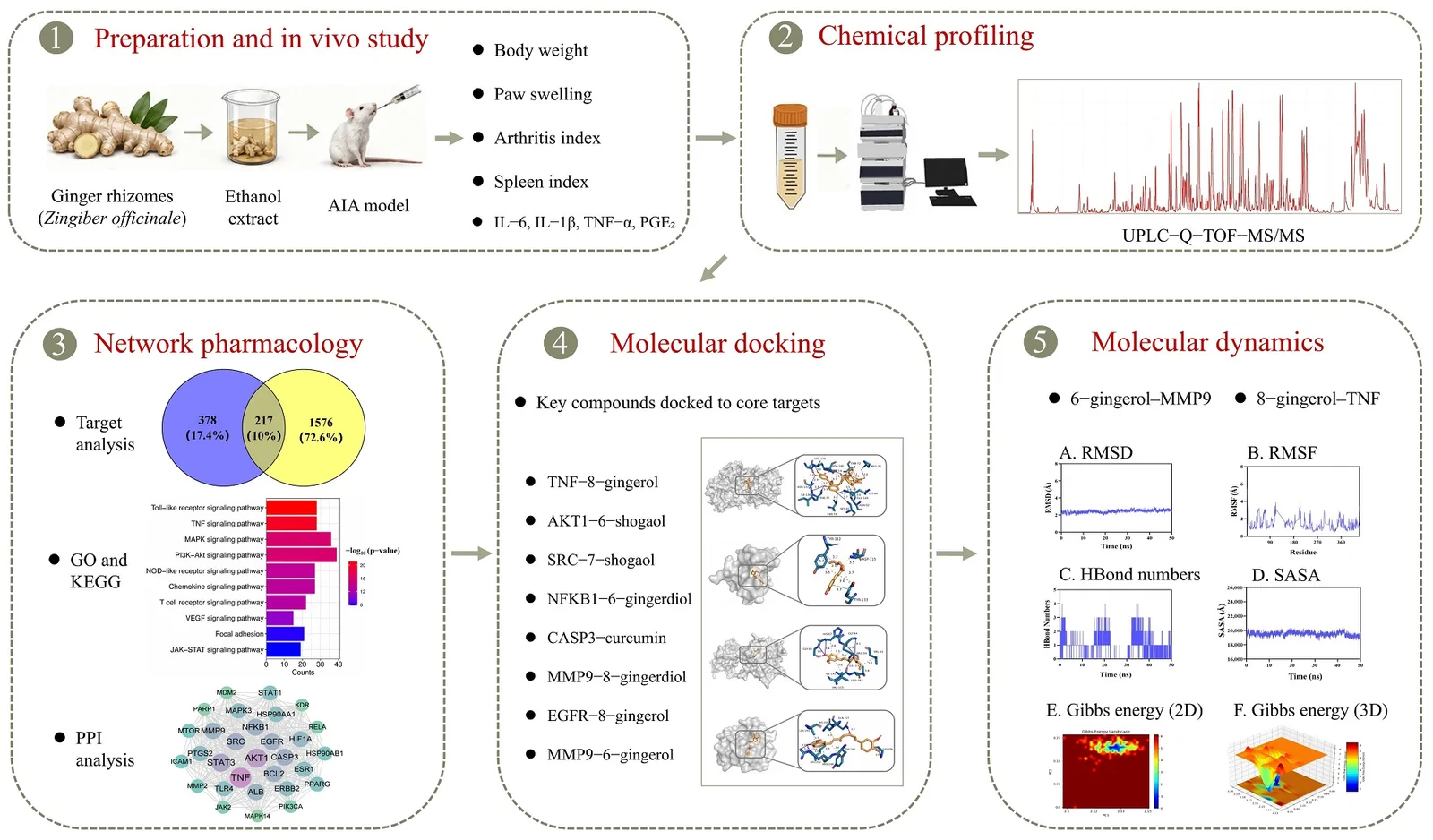
Overall study design. Ginger ethanolic extract (GE) preparation, pharmacodynamic evaluation in adjuvant-induced arthritis (AIA) rats, ultra-performance liquid chromatography–quadrupole time-of-flight tandem mass spectrometry (UPLC-Q-TOF-MS/MS)-based chemical profiling, network pharmacology, molecular docking, and molecular dynamics (MD) simulation were integrated into the workflow. The representative thumbnails only indicate the corresponding analytical steps and were derived from analyses shown in later figures.

Therefore, this study aimed to evaluate the antiarthritic activity of ginger ethanolic extract (GE) in adjuvant-induced arthritis (AIA) rats, characterize GE constituents using ultra-performance liquid chromatography–quadrupole time-of-flight tandem mass spectrometry (UPLC-Q-TOF-MS/MS), and explore potential constituent-target-pathway relationships through network pharmacology, molecular docking, and molecular dynamics (MD) simulation (Figure 1). Because these computational approaches are predictive, the mechanistic findings were interpreted as hypothesis-generating rather than as direct evidence of in vivo target engagement.

## 2. Materials and Methods

### 2.1. Instruments

UPLC-Q-TOF-MS/MS measurements were performed on a Waters platform (Waters Corporation, Milford, MA, USA) consisting of a Xevo G2-XS QTof mass spectrometer with an ESI source, an ACQUITY UPLC I-Class Plus system, and an ACQUITY UPLC BEH C18 column (2.1 mm × 100 mm, 1.7 μm). The supporting apparatus comprised a 5424R refrigerated centrifuge (Eppendorf, Hamburg, Germany), KQ-500DE ultrasonic cleaner (Kunshan Ultrasonic Instruments Co., Ltd., Kunshan, China), N-1300D-WB rotary evaporator (EYELA, Tokyo, Japan), FreeZone 2.5 L freeze dryer (Labconco, Kansas City, MO, USA), Milli-Q IQ 7000 water-purification system (Merck Millipore, Darmstadt, Germany), 37140 paw-volume plethysmometer (Ugo Basile, Gemonio, Italy), and Synergy H1 multimode microplate reader (BioTek, Winooski, VT, USA).

### 2.2. Drugs and Reagents

Fresh rhizomes of *Zingiber officinale* Roscoe were purchased from Qingping Chinese Medicinal Materials Market, Guangzhou, China (batch No. GZ20250318). The material was authenticated by Researcher Xilong Zheng (Guangdong Pharmaceutical University) and was consistent with the macroscopic description of ginger in the 2025 edition of the *Chinese Pharmacopoeia*. The authenticated rhizomes were dried, powdered, and extracted under reflux with 70% ethanol. The filtrate was concentrated under reduced pressure, freeze-dried to obtain ginger ethanolic extract (GE), and stored at 4 °C until use. The medium GE dose was selected by adult-human-to-rat body-surface-area conversion. In the study protocol, the clinical adult crude-drug dose used for conversion was 20 g/day for a 60 kg human adult. The rat equivalent dose was calculated as follows: rat dose (g·kg^−1^·d^−1^) = adult human dose (g/day)/60 kg × (human Km/rat Km), where the Km values for adult humans and rats were 37 and 6, respectively. Thus, 20/60 × 37/6 ≈ 2.05 g·kg^−1^·d^−1^, which was used as the medium dose.

The following materials were used for the pharmacodynamic experiments: MTX (batch No. M7824-1G; Sigma-Aldrich, St. Louis, MO, USA) as the positive control; Freund’s complete adjuvant (FCA; batch No. 240365; Chondrex, Woodinville, WA, USA) for AIA induction; sterile 0.9% saline (batch No. A23091201; Sichuan Kelun Pharmaceutical Co., Ltd., Chengdu, China); sodium carboxymethylcellulose (CMC-Na; batch No. C104985; Shanghai Aladdin Biochemical Technology Co., Ltd., Shanghai, China) to prepare the 0.5% vehicle; and isoflurane (batch No. R510-22; Shenzhen RWD Life Science Co., Ltd., Shenzhen, China) for anesthesia.

Acetonitrile (batch No. 237290; Merck, Darmstadt, Germany), methanol (batch No. 235624; Merck, Darmstadt, Germany), and formic acid (batch No. BCBV8501; Sigma-Aldrich, St. Louis, MO, USA) were liquid chromatography–mass spectrometry (LC-MS) grade and were used for UPLC-Q-TOF-MS/MS analysis. Rat enzyme-linked immunosorbent assay (ELISA) kits for IL-1β (batch No. 20250415), TNF-α (batch No. 20250409), IL-6 (batch No. 20250415), and PGE_2_ (batch No. 20250415) were obtained from Jiangsu Kete Biotechnology Co., Ltd., Yancheng, China. All reagents and kits were used before the corresponding expiration dates.

### 2.3. Experimental Animals

The animals were specific pathogen-free (SPF) male Sprague–Dawley (SD) rats weighing 200 ± 20 g at 6–8 weeks of age. They were obtained from the Laboratory Animal Center of Southern Medical University [Laboratory Animal Production License No. SCXK (Yue) 2021-0041] and maintained in the same center’s SPF barrier facility [Laboratory Animal Use License No. SYXK (Yue) 2021-0167]. Housing conditions were 22 ± 2 °C, 50 ± 10% relative humidity, and a 12 h light/dark schedule. Food and water were provided freely, and a 7-day acclimatization period preceded the experiment.

The protocol was approved by the Experimental Animal Ethics Committee of Southern Medical University (approval No. SMULAC-2025-0318). All operations complied with institutional animal-care regulations and ARRIVE reporting principles. Animal suffering and the numbers of animals used were minimized where possible.

A total of 36 rats were used in the pharmacodynamic experiment (six groups, *n* = 6 per group). The same animals contributed to longitudinal body-weight, paw-volume, and arthritis-index assessments and to endpoint spleen-index and serum inflammatory mediator analyses.

### 2.4. Preparation of GE

Visible impurities were removed from the authenticated ginger. The material was washed, dried at 45 °C to a constant weight, pulverized, and passed through a No. 6 sieve. The powdered ginger (1000 g) was combined with 70% ethanol at a 1:10 (g/mL) ratio, soaked for 60 min, and extracted three times under reflux for 2 h each time. The filtrates were pooled and evaporated at 45 °C under reduced pressure until residual ethanol was no longer detectable by its odor; this was followed by lyophilization to obtain GE. The dry extract was weighed to determine extraction yield.

Yield (%) = [mass of lyophilized GE powder/mass of dried ginger powder] × 100%.

The lyophilized extract was sealed in amber vials and stored at −20 °C. Before administration, GE suspensions for the low-, medium-, and high-dose groups were freshly prepared with 0.5% CMC-Na and vortexed until uniform.

### 2.5. AIA Model Establishment, Grouping, and Treatment [27]

#### 2.5.1. Animal Grouping, Model Induction, and Administration

After acclimatization, SPF male SD rats were stratified by body weight and assigned to six groups (*n* = 6 each): normal control (NC), AIA model control (MC), MTX positive control (PC), low-dose GE (GE-L), medium-dose GE (GE-M), and high-dose GE (GE-H). Except for the NC rats, all animals received a subcutaneous injection of 0.1 mL FCA into the plantar surface of the right hind paw; NC rats received an equal volume of sterile saline at the same site. General condition and paw erythema or swelling were checked daily. On day 7 after injection, marked paw edema, impaired joint movement, and increased AI score indicated successful AIA induction.

Drug administration began on day 7 after model induction and lasted 35 days. NC and MC rats were gavaged with 0.5% CMC-Na. PC rats received MTX at 1.0 mg·kg^−1^ by oral gavage three times per week. GE-L, GE-M, and GE-H rats received 1.03, 2.05, and 4.10 g·kg^−1^·d^−1^ GE, respectively. These doses corresponded to 0.5-, 1-, and 2-fold of the rat equivalent of the adult dose described in Section 2.2. The gavage volume was 10 mL·kg^−1^ and was adjusted weekly according to body weight.

#### 2.5.2. Pharmacodynamic Measurements

Body weight and volume of the inflamed hind paw were measured before FCA or saline injection; immediately before treatment; and on treatment days 7, 14, 21, 28, and 35. For paw-volume measurement, the affected paw was gently immersed into the plethysmometer to the same ankle mark at each assessment. Three measurements were taken for each rat and averaged. Paw swelling was calculated as follows [28,29]:

Paw swelling (mL) = post-modeling paw volume − pre-modeling paw volume

AI scoring started on day 7 after model induction and was repeated every 7 days. Two investigators blinded to group allocation scored the animals independently, and the mean score was used for analysis. The scale was as follows [30]: 0, no erythema or swelling; 1, slight erythema or swelling restricted to the toes or ankle; 2, mild-to-moderate swelling involving the metatarsophalangeal joints; 3, obvious erythema and swelling below the ankle; and 4, severe swelling, deformity, or pronounced limitation of movement involving the whole paw and ankle. Scores from the four limbs were summed for each rat, with a maximum total AI score of 16. After the final administration, rats were fasted for 12 h with free access to water, anesthetized, and euthanized. The spleen was removed and weighed, and body weight immediately before tissue collection was recorded for spleen-index calculation [31].

Spleen index (mg/g) = spleen weight (mg)/pre-collection body weight (g)

#### 2.5.3. Serum Collection and Inflammatory Mediator Assays

At study completion, rats were anesthetized with 2% isoflurane, and 3–5 mL of blood was drawn from the abdominal aorta. Blood samples stood for 60 min at room temperature and were then centrifuged at 3500 rpm for 10 min at 4 °C. The separated serum was kept at −80 °C. IL-1β, TNF-α, IL-6, and PGE_2_ were measured using the commercial ELISA kits listed in Section 2.2. Briefly, standards and serum samples were brought to room temperature, added to antibody-coated wells in duplicate, incubated according to the manufacturer’s instructions, washed to remove unbound material, reacted with enzyme conjugate and chromogenic substrate, terminated with stop solution, and read at 450 nm. Concentrations were calculated from kit-specific standard curves.

### 2.6. UPLC-Q-TOF-MS/MS Analysis of GE Constituents

#### 2.6.1. Preparation of the Analytical Solution

For MS analysis, 50 mg of GE was diluted to 10 mL with 80% methanol in a volumetric flask. After 30 min ultrasonication, the preparation was centrifuged at 12,000 rpm for 10 min at 4 °C. The supernatant was filtered through a 0.22 μm membrane and placed in an autosampler vial.

#### 2.6.2. Chromatographic Conditions

Separation was carried out on an ACQUITY UPLC BEH C18 column (2.1 mm × 100 mm, 1.7 μm). Mobile phase A was water with 0.1% formic acid, and mobile phase B was acetonitrile. The column temperature was 35 °C, the autosampler temperature was 10 °C, the flow rate was 0.3 mL/min, and the injection volume was 2 μL. The elution program was 0–2 min, 5% B; 2–4 min, 5–10% B; 4–7 min, 10–15% B; 7–21 min, 15–30% B; 21–26 min, 30–70% B; 26–28 min, 70–80% B; 28–32 min, 80–95% B; 32–36 min, 95–5% B; and 36–40 min, 5% B.

#### 2.6.3. Mass Spectrometric Conditions

Data were acquired in both positive and negative ESI modes over an *m*/*z* range of 50–1200. The capillary voltage was 3.0 kV in positive mode and 2.5 kV in negative mode. Source temperature and desolvation temperature were 120 °C and 400 °C, respectively, and desolvation and cone gas flows were 800 and 50 L/h, respectively. Low- and high-energy acquisition used collision energies of 6 eV and 20–40 eV. Sodium formate was used for pre-run mass calibration, and leucine enkephalin served as the lock-mass reference.

#### 2.6.4. Constituent Identification

Raw MS files were processed with UNIFI for peak detection, alignment, formula prediction, and MS/MS fragment matching. Compounds were identified or tentatively annotated by integrating retention behavior, accurate mass, adduct or quasi-molecular ions, isotope distribution, diagnostic product ions, comparison with available reference standards, database searches, and reported fragmentation patterns.

### 2.7. Network Pharmacology Analysis

#### 2.7.1. Prediction of Constituent-Related Targets

The constituents annotated by UPLC-Q-TOF-MS/MS were used as chemical inputs. Canonical SMILES strings or SDF records were obtained from PubChem. Putative targets were predicted using SwissTargetPrediction, PharmMapper, and BATMAN-TCM. SwissTargetPrediction was run for Homo sapiens and retained targets with probability > 0. For PharmMapper, the top 300 proteins ranked by normalized fit score were kept. BATMAN-TCM targets were filtered with score cutoff ≥20 and *p* < 0.05. Target names were converted to official gene symbols in UniProt; this was followed by duplicate removal.

#### 2.7.2. Collection of RA-Related Targets

RA-related genes were compiled by querying GeneCards, OMIM, DrugBank, TTD, and DisGeNET with the keyword “rheumatoid arthritis”. GeneCards hits with relevance score ≥ 10 were retained. In DisGeNET, genes within the top 50% of gene–disease association scores were selected. Relevant targets from the remaining databases were added, and the combined list was deduplicated to generate the final disease-target set.

#### 2.7.3. Identification of Intersection Targets and Network Construction

Candidate anti-RA targets were defined as the intersection between ginger constituent-related targets and RA-related disease targets. A compound-target network was generated in Cytoscape 3.10.3. Degree, betweenness centrality, and closeness centrality were used to rank candidate constituents and therapeutically relevant targets.

#### 2.7.4. Gene Ontology (GO) and Kyoto Encyclopedia of Genes and Genomes (KEGG) Enrichment Analyses

GO and KEGG enrichment of the intersecting targets was conducted with the R package clusterProfiler 4.18.1. GO analysis covered biological process (BP), cellular component (CC), and molecular function (MF) categories, while KEGG analysis was used to identify enriched signaling pathways. Enrichment terms with *p* < 0.05 and FDR < 0.05 were considered significant, and the top 20 terms were visualized.

#### 2.7.5. Construction of the Compound–Target–RA Network

In Cytoscape 3.10.3, candidate active constituents, core targets, and RA were merged to form a compound–target–RA network.

#### 2.7.6. Protein–Protein Interaction (PPI) Network Construction and Core-Target Screening

The intersecting targets were uploaded to STRING with Homo sapiens as the species and an interaction confidence threshold of 0.7. The exported TSV interaction file was imported into Cytoscape 3.10.3 for visualization. Core targets were ranked in CytoHubba v1.6+ using the Degree, MCC, Betweenness, and Closeness algorithms.

Candidate pairs were prioritized for docking only after target screening had been completed. Specifically, we considered three criteria: (i) high network centrality or CytoHubba rank among the 217 ginger–RA intersection targets; (ii) biological relevance to RA inflammation, immune regulation, prostaglandin production, or matrix remodeling; and (iii) connectivity with MS-annotated high-degree constituents. Pairs satisfying these criteria were taken forward for docking and MD simulation.

### 2.8. Molecular Docking [32]

Based on the preceding compound–target–RA and PPI analyses, we selected docking ligands from high-degree MS-annotated constituents and selected receptors from the CytoHubba-ranked hub targets with direct relevance to RA-associated inflammatory or immune pathways. This ensured that target prioritization preceded the docking step. PubChem supplied the 3D ligand structures. We minimized their energies in Chem3D 20.0, converted the files with Open Babel 3.1.1, assigned Gasteiger charges and rotatable bonds in AutoDockTools 1.5.7, and saved the ligands in pdbqt format. Protein structures were obtained from the RCSB PDB. In PyMOL 2.6.0, we removed crystallographic water, co-crystallized ligands, and unrelated small molecules; AutoDockTools then added polar hydrogens and charges and generated receptor pdbqt files. AutoDock Vina 1.2.3 performed the docking runs. For each ligand–target pair, we retained the pose with the lowest energy and a chemically reasonable orientation. PyMOL and Discovery Studio Visualizer 2021 were used to inspect hydrogen bonds, hydrophobic contacts, π–π stacking, π–alkyl interactions, and van der Waals contacts.

### 2.9. Molecular Dynamics (MD) Simulation [33,34]

We carried out MD simulations in GROMACS 2022.5 for docked complexes that combined favorable binding energy with a plausible binding pose. Protein topologies used the CHARMM36m force field, and ligand parameters were generated with the CGenFF server. Each complex was centered in a dodecahedral TIP3P water box, neutralized with Na^+^ or Cl^−^ ions, and adjusted to 0.15 mol/L NaCl. After steepest-descent energy minimization, each system underwent 500 ps NVT and 500 ps NPT equilibration at 300 K and 1 bar. We then ran 50–100 ns production simulations with a 2 fs time step and stored trajectories every 10 ps. Root-mean-square deviation (RMSD), root-mean-square fluctuation (RMSF), radius of gyration (Rg), solvent-accessible surface area (SASA), hydrogen-bond number, and Gibbs free energy landscapes were calculated to describe conformational behavior and binding stability.

### 2.10. Data Analysis and Statistics

Animal data were analyzed using GraphPad Prism 9.0 and SPSS 26.0. Quantitative data are expressed as mean ± SD. Normality and homogeneity of variance were assessed before statistical testing. Multiple-group comparisons were performed by one-way analysis of variance. Tukey’s post hoc test was used when variances were homogeneous; when variances were unequal, Welch’s analysis of variance followed by Dunnett’s T3 test was applied. Repeated measures, including body weight, paw swelling, and AI score, were analyzed by two-way repeated-measures analysis of variance. *p* < 0.05 was considered statistically significant.

## 3. Results

### 3.1. GE Attenuated Arthritic Manifestations in AIA Rats

**Figure 2:**
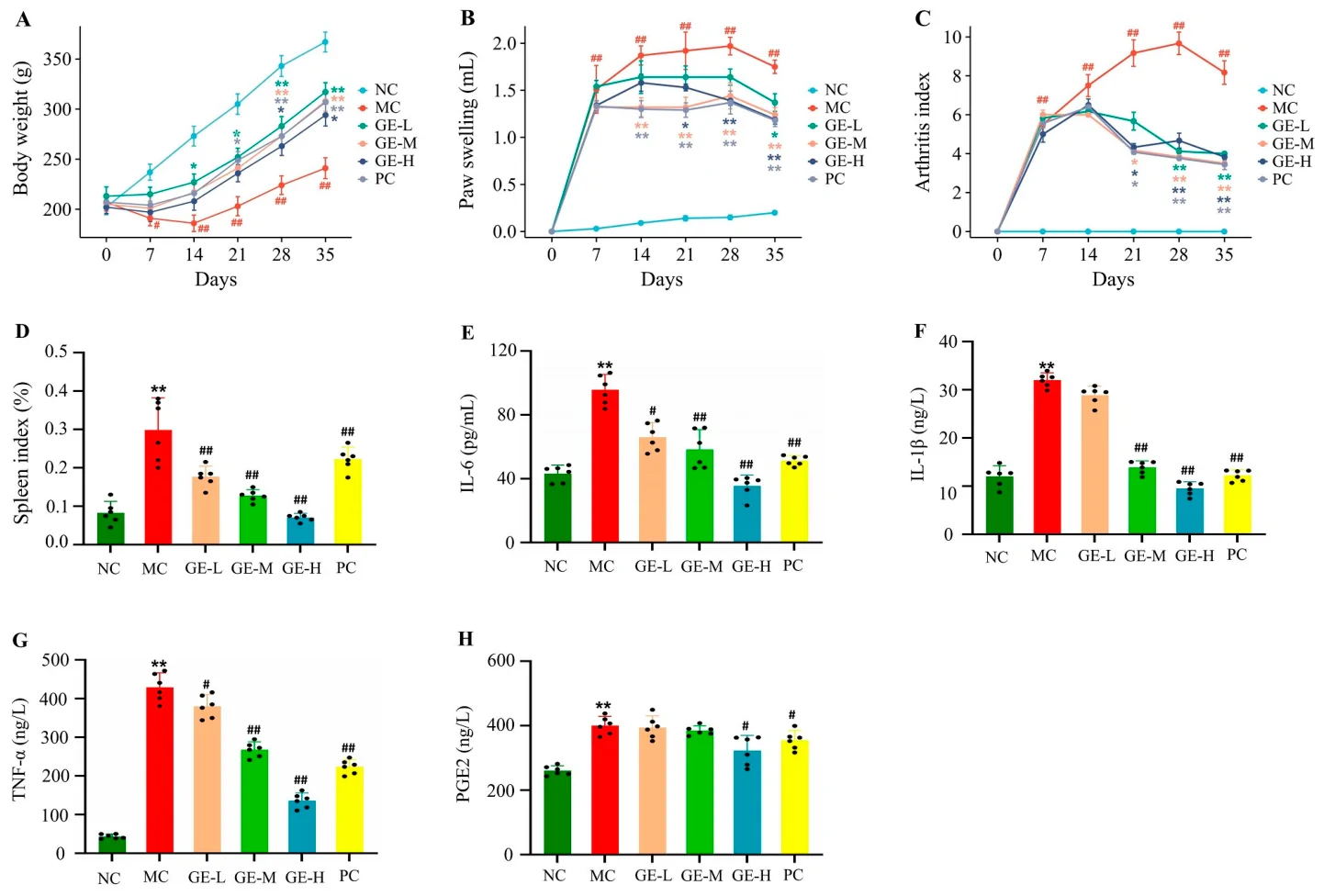
Effects of ginger ethanolic extract (GE) on adjuvant-induced arthritis (AIA) manifestations and serum inflammatory mediators in rats (mean ± SD, *n* = 6 per group). (**A**): body weight; (**B**): paw-swelling dynamics; (**C**): arthritis index (AI) score; (**D**): spleen index; (**E**–**H**): serum interleukin-6 (IL-6), interleukin-1β (IL-1β), tumor necrosis factor-α (TNF-α), and prostaglandin E_2_ (PGE_2_). NC, normal control; MC, AIA model control; GE-L, low-dose GE; GE-M, medium-dose GE; GE-H, high-dose GE; PC, methotrexate (MTX) positive control. # *p* < 0.05 and ## *p* < 0.01 versus NC; * *p* < 0.05 and ** *p* < 0.01 versus MC.

After FCA injection, the MC group developed the expected AIA signs: paw erythema and swelling, less spontaneous activity, poorer general condition, and slower weight gain. MTX was retained as the reference treatment. Compared with NC rats, MC rats gained markedly less weight. GE treatment, especially at the medium and high doses, partially reversed this trend; MTX produced a similar recovery pattern (Figure 2A). This change is consistent with reduced inflammation-related systemic deterioration rather than a purely local effect.

Paw volume and AI score followed the same pattern. In MC rats, both indices increased and remained elevated during observation. GE and MTX reduced paw swelling and AI scores, with clearer responses in the GE-M, GE-H, and PC groups (Figure 2B,C). AIA induction also increased the spleen index, which fell after GE or MTX treatment and was lowest in the GE-H group (Figure 2D). The parallel changes in joint and spleen indices indicate that GE reduced local inflammation and eased AIA-associated immune activation.

In the MC group, serum IL-6, IL-1β, TNF-α, and PGE_2_ were higher than in NC rats, showing that the arthritic phenotype was accompanied by systemic inflammation. GE reduced these four mediators, and the response generally increased with dose. GE-H produced the clearest decreases across all markers. GE-M reduced IL-1β and TNF-α, whereas the fall in PGE_2_ was most evident in the GE-M and PC groups (Figure 2E–H). These biochemical changes paralleled the improvements in paw swelling and AI score.

### 3.2. UPLC-Q-TOF-MS/MS Chemical Profiling of GE

**Figure 3:**
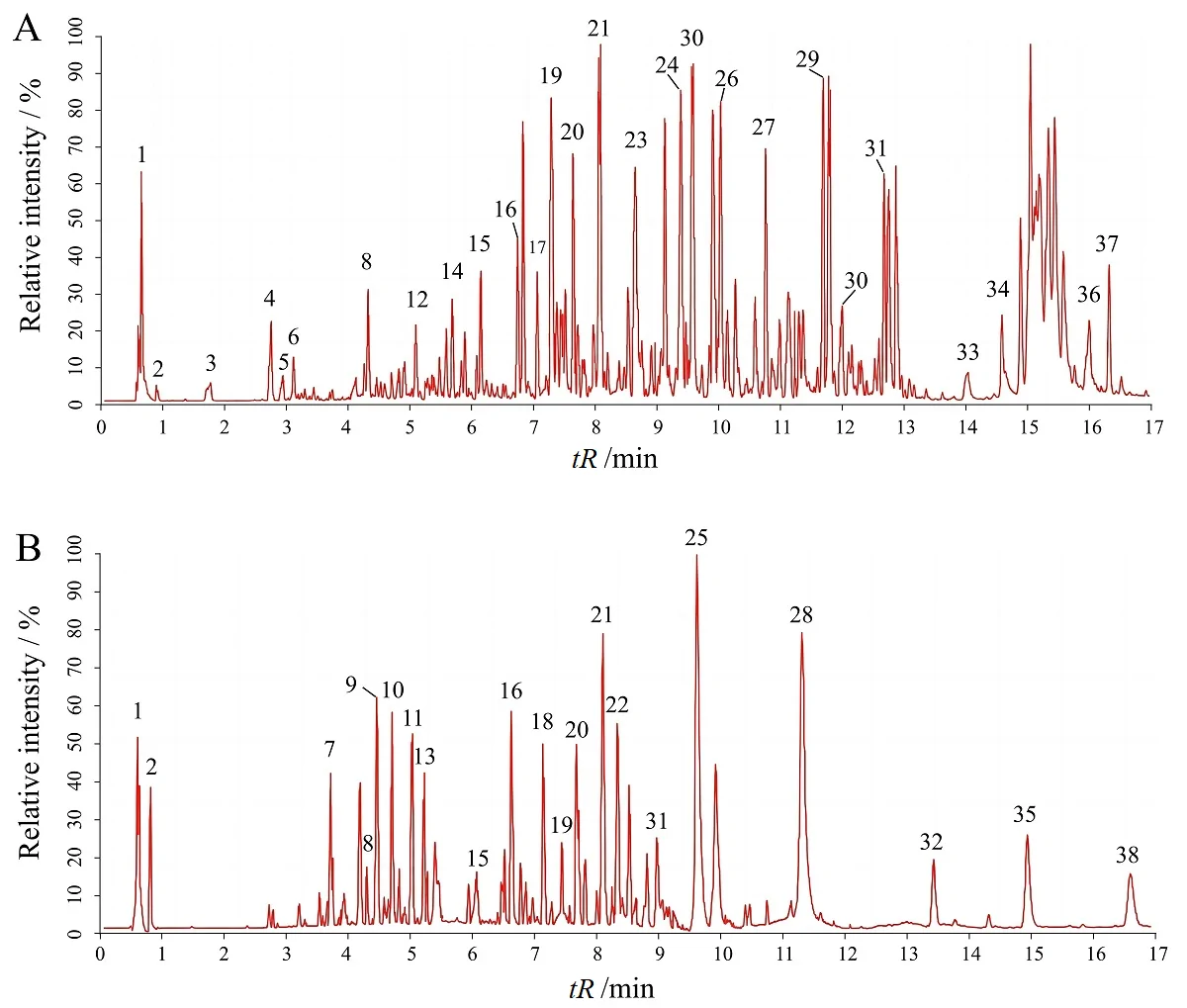
Ginger ethanolic extract (GE) total ion chromatograms acquired by ultra-performance liquid chromatography–quadrupole time-of-flight tandem mass spectrometry (UPLC-Q-TOF-MS/MS). (**A**): positive-ion mode; (**B**): negative-ion mode. The numbers indicate the corresponding compound numbers listed in Table 1. tR, retention time.

GE was analyzed by UPLC-Q-TOF-MS/MS to define its chemical profile. Both positive- and negative-ion chromatograms showed abundant signals, indicating a chemically complex extract (Figure 3). By integrating retention time, accurate mass, quasi-molecular ions, characteristic fragments, database matching, and literature reports, 38 constituents were identified or tentatively annotated (Table 1).

Among the 38 annotations, two were organic acid/amino acid components and the other 36 belonged to phenolic classes. Gingerols, shogaols, gingerdiones, and gingerdiols accounted for much of the profile; gingerones, gingerene-type compounds, and diarylheptanoid/curcuminoid derivatives were also detected. The most notable annotations included 6-, 8-, and 10-gingerol; 6- and 7-shogaol; 6- and 8-gingerdiol; 1-dehydro-6-, -8-, and -10-gingerdione; curcumin; and hexahydrocurcumin. This chemical distribution offers a basis for linking GE to the anti-AIA effects observed in vivo.

### 3.3. Network-Pharmacology Findings

**Figure 4:**
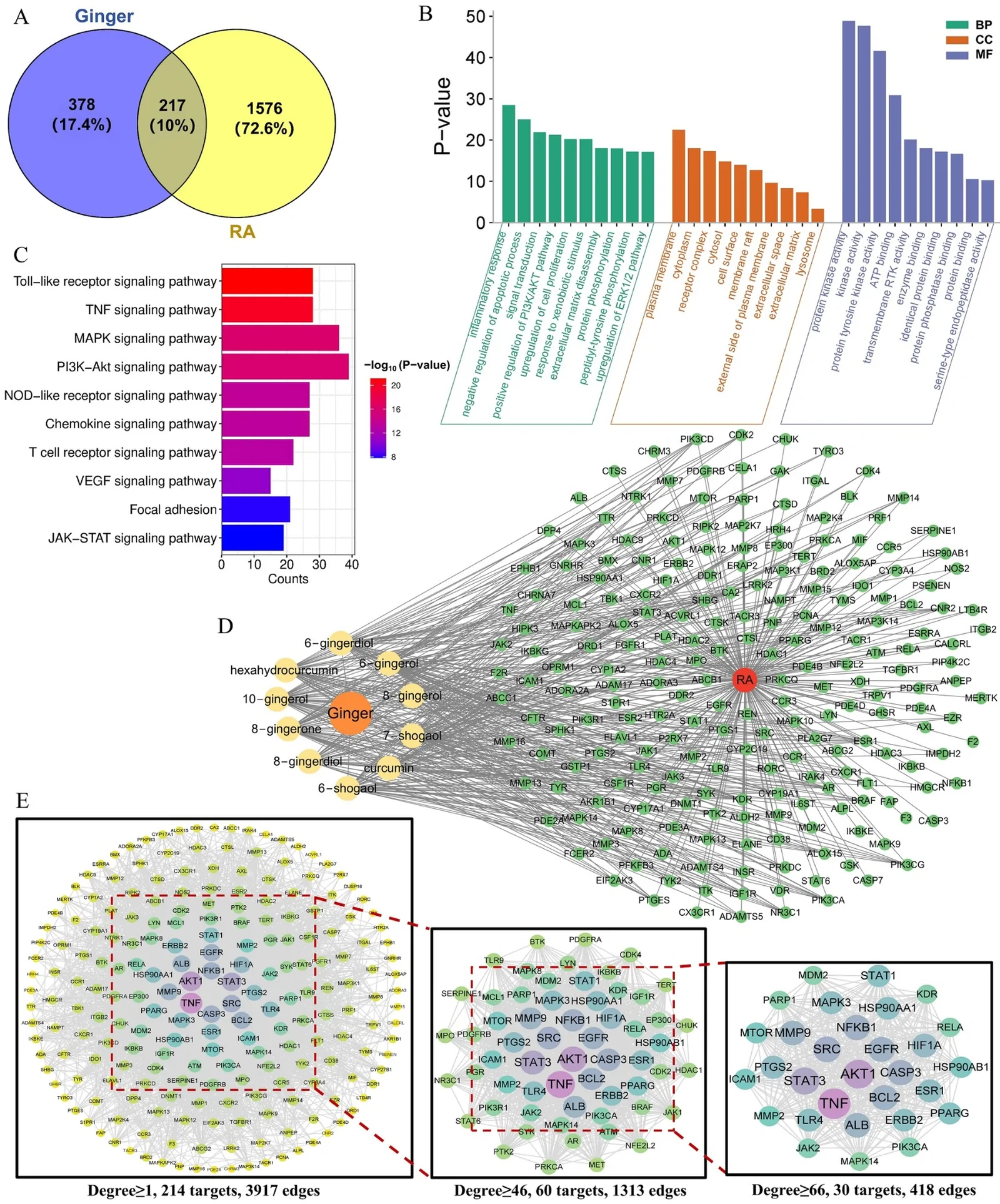
Network-pharmacology analysis of ginger against rheumatoid arthritis (RA). (**A**): Venn overlap between constituent-related targets and RA-related targets; (**B**): Gene Ontology (GO) enrichment; (**C**): Kyoto Encyclopedia of Genes and Genomes (KEGG) enrichment; (**D**): network of ginger-core–constituent-–target network-RA; (**E**): protein–protein interaction (PPI) network and core–target screening.

Submitting the 38 MS-annotated constituents to SwissTargetPrediction, PharmMapper, and TCMSP/BATMAN-TCM yielded 595 nonredundant ginger-related targets after UniProt standardization. GeneCards, OMIM, DrugBank, TTD, and DisGeNET mining returned 1793 RA-related disease targets. Their overlap contained 217 candidate anti-RA targets (Figure 4A). This overlap fits a multicomponent pharmacological profile for ginger, but individual targets still require experimental validation.

GO analysis of the 217 intersecting targets produced 821 BP, 96 CC, and 193 MF terms. The top BP terms clustered around inflammatory responses, extracellular-matrix disassembly, PI3K/AKT activation, ERK1/2 activation, anti-apoptotic regulation, and cell proliferation. The CC terms mapped mainly to receptor complexes, the cell surface, membrane rafts, extracellular matrix, and extracellular space. MF enrichment was dominated by protein tyrosine kinase activity, receptor tyrosine kinase activity, general kinase activity, and enzyme binding (Figure 4B). In this context, the target set links ginger most directly with inflammatory signaling, matrix remodeling, and kinase-dependent cell responses in RA.

KEGG enrichment identified 174 significant pathways. The RA-relevant set included TNF, JAK-STAT, MAPK, T cell receptor, chemokine, TLR, PI3K-Akt, and NLR signaling (Figure 4C). These pathways cover cytokine production, immune-cell activation, synoviocyte expansion, innate immune sensing, and matrix degradation. Their enrichment provides a pathway-level explanation for the lower IL-6, IL-1β, TNF-α, and PGE_2_ levels observed after ginger treatment, although pathway activity requires direct experimental verification.

Degree ranking identified 10 core constituents: hexahydrocurcumin, 8-gingerone, 6-shogaol, curcumin, 7-shogaol, 8-gingerol, 6-gingerol, 6-gingerdiol, 10-gingerol, and 8-gingerdiol. We used these compounds to build the ginger core–constituent/core–target network (Figure 4D). The PPI network for the 217 targets contained 3917 interactions; after stepwise filtering, 30 core targets and 418 edges remained (Figure 4E). TNF, NFKB1, IL6, PTGS2/COX-2, STAT3, MAPK3, AKT1, TLR4, and MMP9 were among the most connected nodes. The result places ginger phenolics at the intersection of cytokine signaling, PTGS2/PGE_2_ production, MAPK/PI3K-Akt/JAK-STAT cascades, and extracellular-matrix remodeling.

### 3.4. Docking Analysis

**Figure 5:**
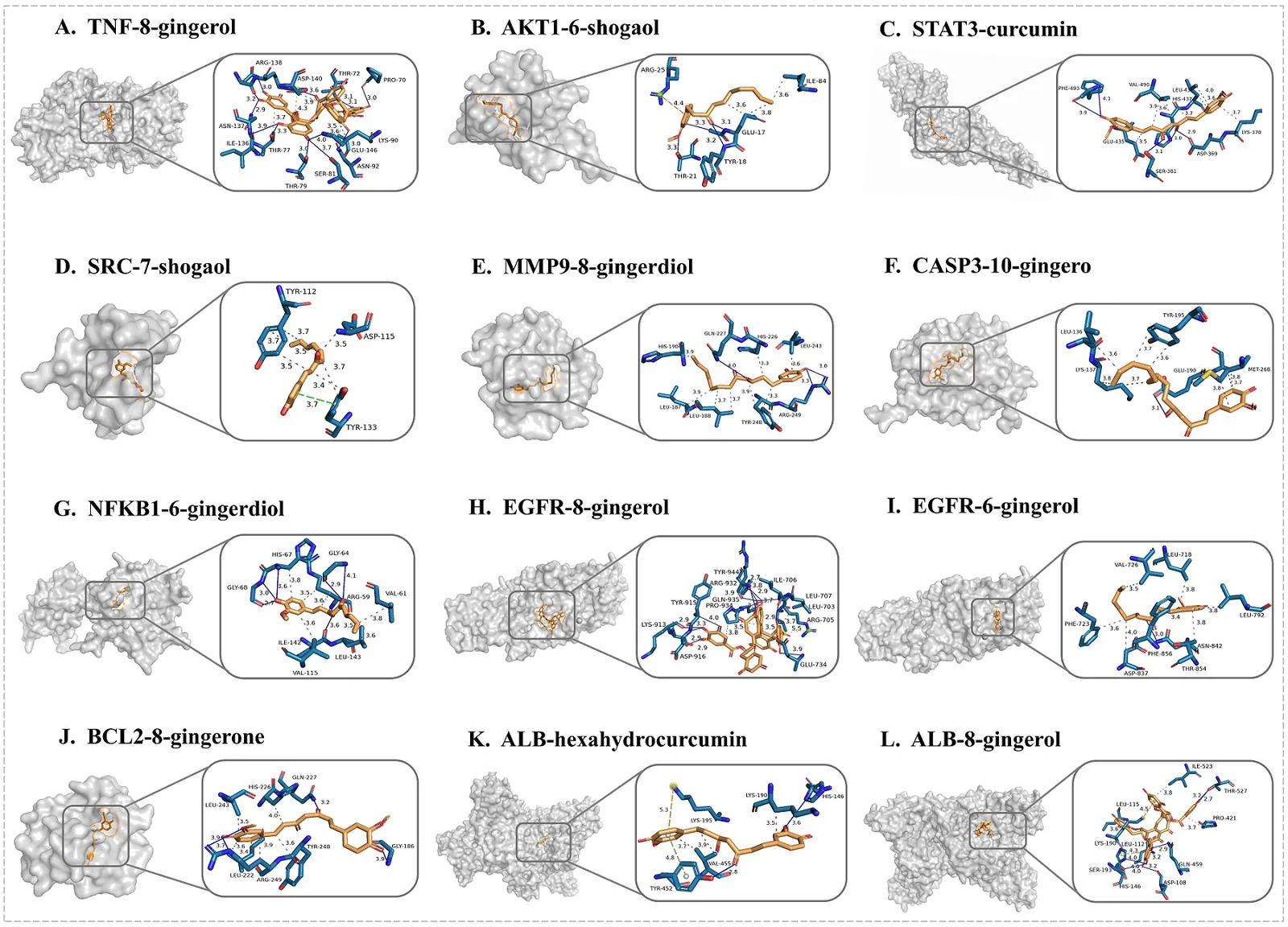
Docking views of representative core constituent–target pairs. (**A**): TNF-8-gingerol; (**B**): AKT1-6-shogaol; (**C**): STAT3-curcumin; (**D**): SRC-7-shogaol; (**E**): MMP9-8-gingerdiol; (**F**): CASP3-10-gingerol; (**G**): NFKB1-6-gingerdiol; (**H**): EGFR-8-gingerol; (**I**): EGFR-6-gingerol; (**J**): BCL2-8-gingerone; (**K**): ALB-hexahydrocurcumin; (**L**): ALB-8-gingerol. In the docking views, blue/cyan sticks represent amino acid residues in the protein binding pocket, yellow/orange sticks represent the docked ligands, and oxygen and nitrogen atoms are shown in red and dark blue, respectively.

We next docked the core constituents against hub targets to examine whether the network pairs had plausible binding modes. The ligand panel included hexahydrocurcumin, 8-gingerone, 6-shogaol, curcumin, 7-shogaol, 8-gingerol, 6-gingerol, 6-gingerdiol, 10-gingerol, and 8-gingerdiol, and the target panel included TNF, AKT1, STAT3, SRC, CASP3, NFKB1, EGFR, BCL2, ALB, and MMP9 (Figure 5). Docking energies ranged from −10.130 to −5.112 kcal/mol (Table 2). The strongest 10 pairs were 8-gingerol-ALB (−10.130 kcal/mol), 8-gingerol-TNF (−9.655 kcal/mol), 8-gingerol-EGFR (−9.217 kcal/mol), curcumin-MMP9 (−9.138 kcal/mol), 8-gingerone-MMP9 (−8.786 kcal/mol), 8-gingerol-MMP9 (−8.770 kcal/mol), 10-gingerol-MMP9 (−8.721 kcal/mol), 6-gingerol-MMP9 (−8.597 kcal/mol), 6-gingerdiol-MMP9 (−8.554 kcal/mol), and 7-shogaol-MMP9 (−8.488 kcal/mol). Strong 8-gingerol binding to TNF and MMP9, together with broad MMP9 affinity for gingerol/shogaol-type molecules, supported the selection of 8-gingerol-TNF and 6-gingerol-MMP9 for MD simulation.

### 3.5. MD Simulation Analysis

**Figure 6:**
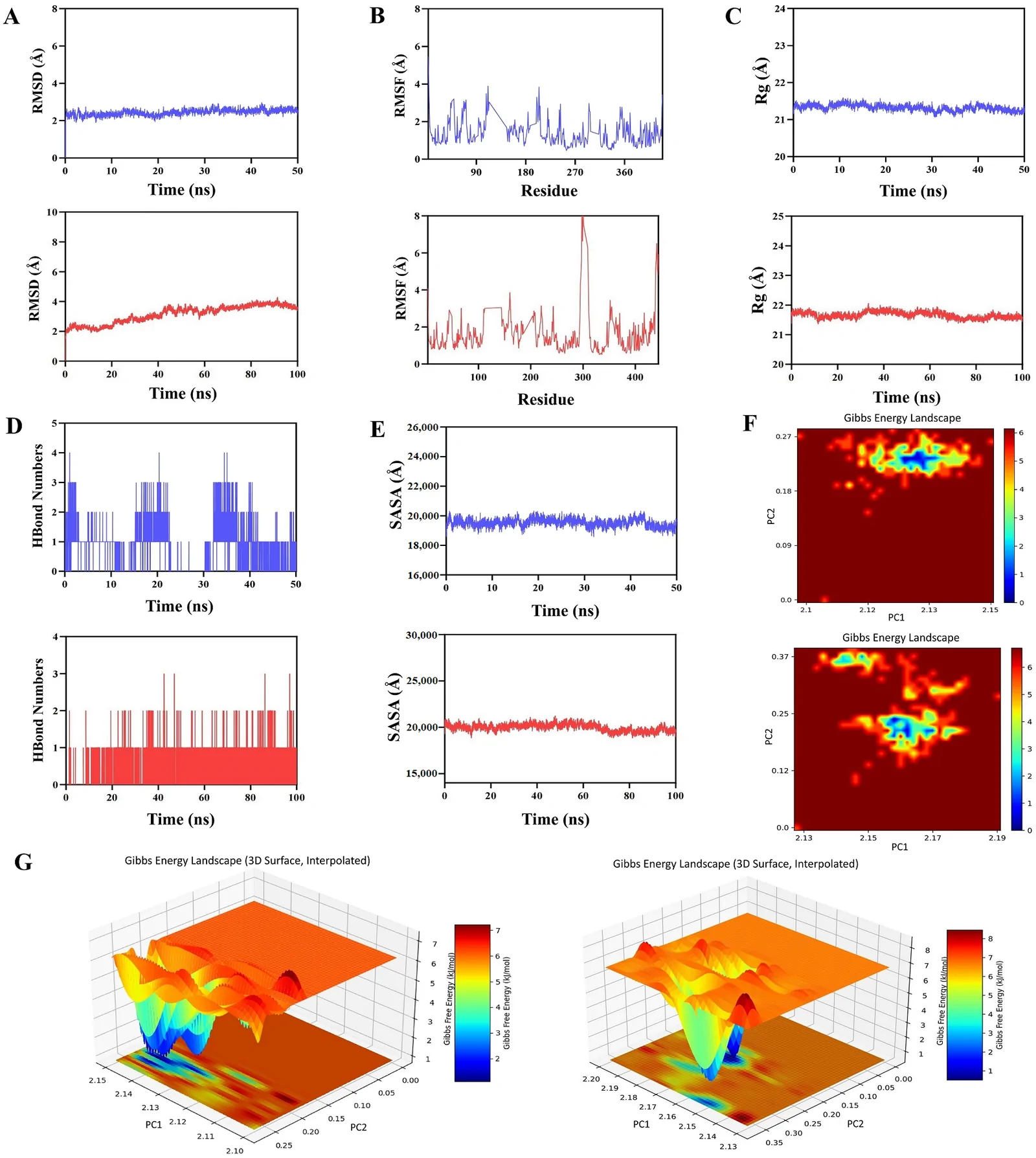
Molecular dynamics (MD) simulation outputs for representative constituent–target complexes. (**A**): protein-backbone root-mean-square deviation (RMSD); (**B**): residue root-mean-square fluctuation (RMSF); (**C**): radius of gyration (Rg); (**D**): hydrogen-bond number; (**E**): solvent-accessible surface area (SASA); (**F**): two-dimensional Gibbs free energy landscape heat map; (**G**): three-dimensional Gibbs free energy landscape surface plot. Upper blue curves/plots (**A**–**F**) or left panels (**G**) correspond to the 8-gingerol-TNF complex; lower red curves/plots (**A**–**F**) or right panels (**G**) correspond to the 6-gingerol-MMP9 complex.

We selected the 8-gingerol-TNF (−9.655 kcal/mol) and 6-gingerol-MMP9 (−8.597 kcal/mol) complexes for MD simulation to test post-docking stability. During the 50 ns trajectory, 8-gingerol-TNF maintained a limited backbone RMSD range of approximately 2.0–2.8 Å. The 6-gingerol-MMP9 complex adjusted early in the 100 ns simulation and then stabilized, with RMSD values mostly around 3.0–4.0 Å. Neither system showed obvious ligand dissociation (Figure 6A).

Most residues remained stable in the RMSF analysis, with larger fluctuations restricted mainly to terminal regions and flexible loops (Figure 6B). Rg values changed little, remaining at approximately 21.2–21.5 Å for 8-gingerol-TNF and 21.5–21.9 Å for 6-gingerol-MMP9, which indicates preserved compactness (Figure 6C). Hydrogen-bond analysis showed intermittent formation of 1–4 bonds in the 8-gingerol-TNF system and 1–3 bonds in the 6-gingerol-MMP9 system (Figure 6D). SASA varied moderately and did not suggest substantial unfolding or exposure of the hydrophobic core (Figure 6E).

The free energy landscapes displayed compact low-energy basins for both complexes, indicating that each system sampled relatively stable conformations during simulation (Figure 6F,G). Overall, the MD outputs supported stable behavior of the 8-gingerol-TNF and 6-gingerol-MMP9 complexes. This structural data support a putative model in which gingerol-related constituents may participate in TNF-related cytokine regulation and MMP9-linked matrix remodeling, but direct in vivo target engagement remains to be verified.

## 4. Discussion

The FCA-induced AIA model reproduced major RA-like manifestations, including impaired body-weight gain, paw edema, increased AI scores, spleen enlargement, and elevated serum IL-6, IL-1β, TNF-α, and PGE_2_. GE reversed these changes to varying degrees, particularly in the medium- and high-dose groups. Rather than interpreting these endpoints as direct proof of structural joint protection, we view them as preclinical evidence that GE attenuates systemic inflammation and clinical arthritis severity in this model. This interpretation is consistent with the established contribution of IL-6, IL-1β, and TNF-α to synovial inflammation and tissue injury [35,36].

UPLC-Q-TOF-MS/MS annotated 38 constituents in GE, with phenolic compounds, including gingerols, shogaols, gingerdiones, and gingerdiols, as the dominant group. Network analysis then connected these compounds with 217 RA-related targets. Enrichment around inflammatory response, matrix disassembly, kinase signaling, proliferation, apoptosis, TNF, JAK-STAT, MAPK, PI3K-Akt, Toll-like receptor (TLR), and nucleotide-binding oligomerization domain-like receptor (NLR) pathways aligns with the serum cytokine reductions observed here and with prior reports in inflammatory and arthritis-related models [37,38]. Nonetheless, network pharmacology identifies plausible associations rather than measured target engagement; therefore, TNF, NFKB1, IL6, PTGS2, STAT3, MAPK3, AKT1, TLR4, and MMP9 should be interpreted as prioritized candidate nodes.

**Figure 7:**
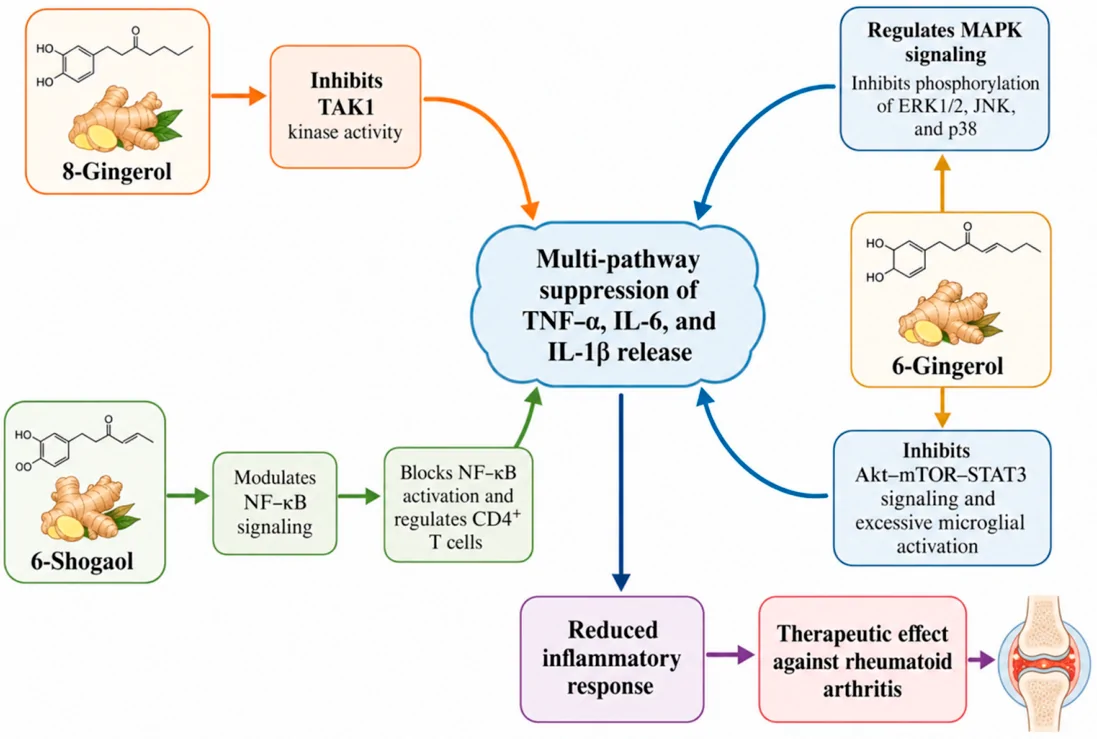
Proposed hypothesis-generating model illustrating how ginger constituents may attenuate rheumatoid arthritis (RA)-related inflammatory mediator release. 6-Gingerol, 8-gingerol, 6-shogaol, and related phenolic constituents may regulate inflammatory responses through multiple targets and signaling pathways. 6-Gingerol may suppress extracellular signal-regulated kinase 1/2 (ERK1/2), c-Jun N-terminal kinase (JNK), and p38 phosphorylation in the mitogen-activated protein kinase (MAPK) pathway and modulate protein kinase B (AKT)–mammalian target of rapamycin (mTOR)–signal transducer and activator of transcription 3 (STAT3) signaling. In the present study, 8-gingerol was computationally prioritized as a putative tumor necrosis factor (TNF)-interacting constituent; however, direct tissue-level validation of the 8-gingerol-TNF interaction remains necessary. 6-Shogaol and related ginger phenolics may regulate nuclear factor-kappa B (NF-κB)-associated signaling in immune cells or synovial cell contexts. These potential effects may contribute to decreased levels of interleukin-6 (IL-6), interleukin-1β (IL-1β), TNF, and other pro-inflammatory mediators, thereby attenuating inflammatory cascades and joint injury. The schematic was created with BioRender (https://www.biorender.com/).

These findings are summarized as a hypothesis-generating model rather than a confirmed signaling cascade (Figure 7). The literature points to several relevant inflammatory axes that may be relevant. For example, 6-Gingerol has been reported to suppress p65, p38, and JNK activation and to reduce IL-1β, IL-6, and TNF-α release in lipopolysaccharide-stimulated RAW264.7 macrophages [39]. It has also been linked to attenuation of Akt-mTOR-STAT3 signaling in microglia [40]. Additional gingerol studies in non-RA inflammatory injury models have reported regulation of PI3K/Akt/mTOR, NF-κB, and RUNX1/NF-κB-related pathways [41,42,43], supporting biological plausibility but not substituting for RA-tissue validation. Broader studies and reviews support anti-inflammatory and immunomodulatory activities for gingerols and shogaols [22,23]. These observations are directionally consistent with the cytokine changes observed in the AIA model, although direct validation in synovial tissue is still needed.

The docking and MD results helped narrow the network predictions to interactions with plausible structural support. Two pairs, 8-gingerol-TNF and 6-gingerol-MMP9, stood out because they combined favorable docking energies with direct relevance to RA biology. During the 50 ns simulation, the 8-gingerol-TNF complex maintained acceptable RMSD variation. The 6-gingerol-MMP9 complex stabilized after its early adjustment phase in the 100 ns trajectory. RMSF, Rg, hydrogen-bond number, SASA, and free-energy landscape results did not contradict stable complex formation. Since TNF amplifies inflammatory signaling and MMP9 contributes to matrix breakdown, synovial invasion, and joint damage [44,45], these two interactions provide plausible entry points for future experimental testing of the broader TNF/MAPK/PI3K-Akt/JAK-STAT/NF-κB-related mechanism. These computational results should not be interpreted as proof of in vivo binding or target inhibition.

When compared with earlier ginger-based arthritis studies, our results are consistent with reports that gingerol-containing *Zingiber officinale* extracts reduce joint inflammation in streptococcal cell wall (SCW)-induced arthritis [46]. Studies of purified ginger constituents add target-level context: 8-shogaol inhibited TAK1-dependent inflammatory signaling and reduced AIA pathology [47], whereas 6-shogaol suppressed rheumatoid arthritis fibroblast-like synoviocyte (RA FLS) proliferation, migration, cytokine/MMP release, and PI3K/AKT/NF-κB signaling [48]. A recent report on oral ginger-derived extracellular vesicles further showed suppression of inflammatory genes in RA synovial fibroblasts and reduced arthritis severity and histological joint damage in mice [49]. These studies emphasize why tissue histology and target-protein validation are important next steps for our ginger framework.

We used MS-detected constituents, rather than an unfiltered database list, as the entry point for target prediction. This choice improves the biological relevance of the network analysis. Combining chemical profiling, network pharmacology, docking, MD simulation, and the AIA rat experiment also gives the study an evidence chain that is stronger than database prediction alone. However, several limitations should be considered when interpreting the results. First, the animal efficacy assessment was limited to macroscopic arthritis indices, spleen index, and serum inflammatory mediators. In this completed AIA experiment, joint tissues suitable for histology, immunohistochemistry, or Western blotting were not retained; therefore, synovial hyperplasia, inflammatory-cell infiltration, cartilage erosion, and bone destruction could not be evaluated by hematoxylin and eosin (H/E), safranin O, tartrate-resistant acid phosphatase (TRAP), or related staining. Second, the predicted hub targets and pathways were not validated in synovium or paw tissue by Western blotting, immunohistochemistry, RT-qPCR, targeted proteomics, or loss- and gain-of-function assays. Thus, the TNF, MMP9, STAT3, AKT1, PTGS2, TLR4, and NF-κB-related assignments should be viewed as mechanistic hypotheses generated from MS-informed network pharmacology, molecular docking, MD simulation, serum mediator changes, and consistency with the literature rather than direct proof of tissue-level target engagement. Third, some compound annotations are tentative and require confirmation and absolute quantification with reference standards. Fourth, systemic exposure, pharmacokinetics, and tissue distribution of the major ginger constituents were not measured. Fifth, only male rats and one AIA model were used, which limits generalizability to sex-dependent responses and other RA models. Future work should integrate joint histopathology, target-protein validation, synovial-cell assays, serum pharmacochemistry, pharmacokinetic/exposure profiling, and formal dose–response/toxicity assessment to identify the principal active constituents and mechanisms more precisely.

## 5. Conclusions

This work chemically profiled GE by UPLC-Q-TOF-MS/MS and evaluated its antiarthritic activity in AIA rats, followed by network, docking, and MD analyses. GE reduced paw swelling, AI scores, spleen index, and serum inflammatory mediators. The chemical and computational results prioritize phenolic constituents, especially gingerols and shogaols, and candidate inflammatory pathways involving TNF, MAPK, PI3K-Akt, and JAK-STAT signaling. Overall, the findings provide an exploratory constituent–target–pathway-efficacy framework rather than direct proof of tissue-level target engagement. They support further validation of ginger-derived constituents as anti-inflammatory leads or adjunct candidates for RA intervention.

## Figures and Tables

**Table 1:** Major constituents of ginger ethanolic extract (GE) identified or tentatively annotated by ultra-performance liquid chromatography–quadrupole time-of-flight tandem mass spectrometry (UPLC-Q-TOF-MS/MS). tR, retention time; ppm, parts per million.

No	tR/ min	Formula	Measured Mass	Calculated Mass	δ/ ppm	Ion Mode	Fragment Ions (*m*/*z*)	Identification Results
1	0.58	C_22_H_28_O_7_	427.1714/403.1753	427.1733/403.1757	−4.4/−1.0	[M+Na]^+^/[M-H]^−^	193.0851, 167.0695, 137.0586/160.8410	2-(4-hydroxy-3,5-dimethoxyphenyl)-6-(4-hydroxy-3-methoxyphenethyl)tetrahydro-2H-pyran-4-ol
2	0.83	C_21_H_26_O_6_	397.1602/373.1648	397.1627/373.1651	−6.3/−0.8	[M+Na]^+^/[M-H]^−^	177.0901, 137.0593, 117.0690/179.0703	hexahydrocurcumin
3	1.75	C_9_H_8_O_3_	165.0552	165.0552	0.0	[M+Na]^+^	182.0321, 165.2145, 136.7845, 123.1458, 91.2241, 205.3247, 188.1247, 146.1478, 118.2210	Coumaric acid
4	2.79	C_11_H_12_N_2_O_2_	205.0969	205.0977	−3.9	[M+Na]^+^	475.0145, 313.0148, 259.2578, 137.2148	L-Tryptophan
5	2.95	C_21_H_27_O_7_	391.1754	391.1733	5.4	[M+Na]^+^	415.1248, 391.2149, 373.2579, 179.2471, 137.2487	1,5-epoxy-3-hydroxy-(3,4-dihydromethoxyphenyl)-7-(4-hydroxy-3-methoxyphenyl) heptane
6	3.08	C_20_H_24_O_7_	375.1434	375.1444	−2.7	[M+Na]^+^	347.1492, 165.0544, 150.0306	5-(6-(3,4-dihydroxyphenethyl)-4-hydroxytetrahydro-2H-pyran-2-yl)-3-methoxybenzene-1,2-diol
7	3.81	C_22_H_30_O_7_	407.206	407.2046	3.4	[M-H]^−^	429.1478, 396.9875, 371.4061, 297.1473, 193.8524, 137.9634	3,5-dihydroxy-1-(4-hydroxy-3,5-dimethoxyphenyl)-7-(4-hydroxy-3-methoxyphenyl) heptane
8	4.35	C_21_H_26_O_7_	413.1552/389.1596	413.1576/389.1600	−5.8/−1.0	[M+Na]^+^/[M-H]^−^	179.0695, 153.0540, 147.0431, 137.0591/359.1489	5-(4-hydroxy-6-(4-hydroxy-3-methoxyphenethyl)tetrahydro-2H-pyran-2-yl)-3-methoxybenzene-1,2-diol
9	4.41	C_20_H_26_O_6_	361.1648	361.1651	−0.8	[M-H]^−^	197.8074, 160.8014, 135.0439	4-(3,5-dihydroxy-7-(4-hydroxy-3-methoxyphenyl)heptyl)benzene-1,2-diol
10	4.81	C_21_H_26_O_7_	389.1588	389.1600	−3.1	[M-H]^−^	209.0828, 195.8094, 179.0689	5-hydroxy-1-(3,4-dihydroxy-5-methoxyphenyl)-7-(4-hydroxy-3-methoxyphenyl)-3-heptanone
11	5.01	C_23_H_28_O_7_	417.1921	417.1913	1.9	[M-H]^−^	460.1476, 417.1478, 357.5489, 179.7801, 137.0124, 115.1087, 91.2145	3,5-diacetoxy-1-(3,4-dihydroxyphenyl)-7-(4-hydroxyphenyl) heptane
12	5.08	C_25_H_32_O_9_	477.2125	477.2125	0.0	[M+Na]^+^	477.2478, 417.1435, 357.1478, 277.1452, 193.3247, 179.4596, 137.4238	3,5-diacetoxy-1-(4-hydroxy-3-methoxyphenyl)-7-(3,4-dihydroxyphenyl) heptane
13	5.25	C_19_H_26_O_4_	341.1718	341.1729	−3.2	[M+Na]^+^	277.1120, 259.1055, 177.0954, 137.0595	1-dehydro-8-gingerdione
14	5.72	C_19_H_28_O_4_	338.2311	338.2331	−5.9	[M+NH_4_]^+^	261.1199, 177.0853, 163.0712, 137.0597, 131.0495	8-gingerone
15	6.12	C_23_H_30_O_8_	457.1824/433.1856	457.1838/433.1862	−3.1/−1.4	[M+Na]^+^/[M-H]^−^	357.1683, 217.1216, 179.0695, 137.0593/373.1639	1-(3,4-dihydroxy-5-methoxyphenyl)-5-hydroxy-7-(4-hydroxy-3-methoxyphenyl)heptan-3-yl acetate
16	6.79	C_25_H_32_O_10_	515.1885/491.1918	515.1893/491.1917	−1.6/0.2	[M+Na]^+^/[M-H]^−^	433.1857, 373.1644, 179.0694/431.1702, 371.1489	3,5-diacetoxy-1,7-bis(3,4-dihydroxy-5-methoxyphenyl)heptane
17	7.09	C_17_H_24_O_3_	277.1783	277.1804	−7.6	[M+H]^+^	177.0916, 137.0603	6-shogaol
18	7.21	C_21_H_20_O_6_	367.1185	367.1182	0.8	[M-H]^−^	298.2169	curcumin
19	7.45	C_23_H_28_O_8_	455.1671/431.1706	455.1682/431.1706	−2.4/0.0	[M+Na]^+^/[M-H]^−^	313.1427, 203.1061, 149.0599, 123.0438/371.1480, 311.1279	1,7-bis(3,4-dihydroxyphenyl)heptane-3,5-diyl-diacetate
20	7.73	C_21_H_24_O_6_	395.1458/371.1498	395.1471/371.1495	−3.3/0.8	[M+Na]^+^/[M-H]^−^	177.0896, 137.0593/160.8411	5-hydroxy-1,7-bis(4-hydroxy-3-methoxyphenyl)hept-4-en-3-one
21	8.08	C_25_H_32_O_9_	499.1942/475.1969	499.1944/475.1968	−0.4/0.2	[M+Na]^+^/[M-H]^−^	417.1898, 357.1686, 179.0700/415.1752, 355.1531, 340.1301	3,5-diacetoxy-7-(4-dihydroxy-3-methoxyphenyl)-1-(3,4-dihydroxy-5-methoxyphenyl)heptane
22	8.47	C_17_H_22_O_4_	313.1400/289.1439	313.1416/289.1440	−5.1/−0.3	[M-H]^−^	177.0544, 145.0287, 117.0339/-	1-dehydro-6-gingerdione
23	8.75	C_18_H_26_O_3_	291.1945	291.1960	−5.2	[M+H]^+^	137.0596	7-shogaol
24	9.46	C_19_H_30_O_4_	345.2028	345.2042	−4.1	[M+Na]^+^	137.0603	8-gingerol
25	9.86	C_19_H_26_O_4_	341.1721/317.1758	341.1729/317.1753	−2.3/1.6	[M-H]^−^	319.1914, 177.0502, 137.0606/-	1,4-didehydro-8-gingerol
26	10.08	C_21_H_32_O_6_	403.2109	403.2097	3.0	[M+Na]^+^	321.2050, 261.1849, 245.0608	Diacetoxy-6-gingerdiol
27	10.91	C_17_H_22_O_4_	291.1589	291.1596	−2.4	[M+H]^+^	273.1482, 241.1206, 177.0548, 145.0284	1-dehydro-6-gingerdione isomer
28	11.45	C_21_H_32_O_5_	387.2146	387.2147	−0.3	[M-H]^−^	287.1901, 179.0681, 137.0595	Acetoxy-8-gingerol
29	11.89	C_21_H_30_O_4_	347.2217	347.2222	−1.4	[M+H]^+^	271.1683, 246.0191, 192.0714, 177.0544, 145.0287, 113.3766	1-dehydro-10-gingerdione
30	12.02	C_21_H_30_O_3_	331.2264	331.2273	−2.7	[M+H]^+^	137.0604	8-paradoldiene
31	12.87	C_17_H_26_O_4_	293.1783	293.1753	10.2	[M+H]^+^	145.9279	6-gingerol
32	13.62	C_23_H_36_O_6_	431.2407	431.2410	−0.7	[M-H]^−^	289.2156, 177.0896, 137.0599	8-gingerdiol
33	14.02	C_17_H_28_O_4_	319.1907	319.1885	6.9	[M+Na]^+^	279.0913, 261.0472, 177.0547, 163.0776	6-gingerdiol
34	14.87	C_23_H_36_O_5_	415.2462	415.2460	0.5	[M+Na]^+^	375.2424, 193.0800, 179.0708, 137.0598	acetoxy-10-gingerol
35	15.04	C_23_H_34_O_4_	375.2524/373.2365	375.2535/373.2379	−2.9/−3.8	[M-H]^−^	183.1795, 177.0545, 137.0598/-	1-dehydro-12-gingerdione
36	16.06	C_21_H_30_O_3_	331.2270	331.2273	−0.9	[M+H]^+^	137.0601	8-paradoldiene isomer
37	16.41	C_21_H_30_O_4_	347.2234	347.2222	3.5	[M+H]^+^	329.2104, 297.1834, 177.0545, 135.1159	1,4-didehydro-10-gingerol
38	16.87	C_21_H_34_O_4_	373.2368	373.2355	3.5	[M-H]^−^	333.1721, 177.0546, 137.0594, 117.0331	10-gingerol

**Table 2:** Docking energies of core constituents with key targets. Values are shown in kcal/mol; Protein Data Bank identifiers (PDB IDs) indicate the protein structures used for each target.

Component/ Target (PDB ID)	TNF (5TSW)	AKT1 (7MYX)	STAT3 (6NJS)	SRC (3EAC)	CASP3 (4QUJ)	NFKB1 (1SVC)	EGFR (4WD5)	BCL2 (5UUK)	ALB (1AO6)	MMP9 (6ESM)
Hexahydrocurcumin	−7.106	−5.979	−6.247	−5.729	−5.231	−5.980	−6.503	−5.725	−6.996	−8.433
8-gingerone	−6.794	−5.853	−6.715	−5.882	−6.669	−6.744	−7.724	−6.800	−7.605	−8.786
6-shogaol	−5.690	−5.551	−5.112	−5.314	−5.892	−5.499	−6.366	−5.946	−6.341	−7.817
Curcumin	−7.678	−5.937	−6.985	−6.404	−7.098	−6.515	−7.112	−7.410	−8.026	−9.138
7-shogaol	−6.244	−5.647	−6.259	−5.986	−6.666	−5.832	−7.749	−6.944	−7.579	−8.488
8-gingerol	−9.655	−6.843	−8.109	−7.260	−7.482	−6.974	−9.217	−8.396	−10.130	−8.770
6-gingerol	−6.438	−5.648	−6.554	−5.577	−6.544	−6.377	−7.052	−6.767	−7.314	−8.597
6-gingerdiol	−6.428	−5.894	−6.553	−5.495	−6.464	−6.878	−7.607	−6.527	−7.170	−8.554
10-gingerol	−6.693	−5.479	−6.586	−5.666	−5.855	−6.531	−7.856	−6.849	−7.806	−8.721
8-gingerdiol	−6.396	−5.636	−6.210	−5.517	−5.532	−6.412	−6.343	−6.772	−7.767	−7.383

## Data Availability

Data supporting the findings of this study are available from the corresponding authors upon reasonable request.

## Abbreviations

The following abbreviations are used in this manuscript:

AI	arthritis index
AIA	adjuvant-induced arthritis
BP	biological process
CC	cellular component
FCA	Freund’s complete adjuvant
GE	ginger ethanolic extract
GO	Gene Ontology
KEGG	Kyoto Encyclopedia of Genes and Genomes
MD	molecular dynamics
MF	molecular function
MTX	methotrexate
PDB IDs	Protein Data Bank identifiers
PPI	protein-protein interaction
RA	rheumatoid arthritis
Rg	radius of gyration
RMSD	root-mean-square deviation
RMSF	root-mean-square fluctuation
SASA	solvent-accessible surface area
TCM	traditional Chinese medicine
UPLC-Q-TOF-MS/MS	ultra-performance liquid chromatography-quadrupole time-of-flight tandem mass spectrometry
